# Identifying early signs of Treatment Resistance in First Episode Psychosis to revise and aid further treatment

**DOI:** 10.1192/j.eurpsy.2023.956

**Published:** 2023-07-19

**Authors:** K. F. Wold, C. B. Flaaten, G. Åsbø, L. Widing, I. Melle

**Affiliations:** 1 Institute of clinical medicine; 2 Department of Psychology, University of Oslo; 3 Research and innovation, Oslo University Hospital, Oslo, Norway

## Abstract

**Introduction:**

Approximately 1/3 of patients with first episode psychosis (FEP) will not benefit from antipsychotic medications and are considered treatment resistant (TR). TR is currently defined as sustained lack of remission with functional loss in the context of two adequate trials of different antipsychotics. Studies suggest that early initiation of clozapine treatment support a better course of illness in TR. Most treatment guidelines recommend clozapine after two antipsychotic failures. In practice, increased dosages of other antipsychotics or polypharmacy are tried out first. Identifying early signs of TR and revising treatment is thus important. Since the TR definition requires adequate lengths of treatment attempts, they are difficult to apply in FEP.

**Objectives:**

The aim of the current study is to 1) investigate if a shorter observation period can be used to identify subgroups of FEP patients with early signs of TR (no indication of early clinical recovery - NoECR) and 2) investigate differences in antipsychotic treatments over the first year compared to patients in full or partial early recovery (ECR/ partial ECR).

**Methods:**

Participants 18 to 65 years in their first year of treatment were recruited from major hospitals in Oslo. The participants met the DSM-IV criteria for schizophrenia, schizophreniform disorder, schizoaffective disorder, and psychotic disorder NOS. A total of 387 completed baseline clinical assessments and 207 one-year follow-up. The SCID-I for DSM-IV was used for diagnosis, symptoms were measured with the SCI-PANSS. Treatment history was gathered through interviews and medical charts. No-ECR was defined as a) Not meeting remission criteria for at least 12 weeks at follow-up, and b) Not regained functioning, i.e., a GFS score < 60. ECR was defined as a) Meeting the criteria for remission and b) Regained functioning, i.e., a GFS score >=61. Partial ECR did not meet these criteria.

**Results:**

At one year follow-up, 47% met the criteria for no-ECR, 29% the criteria for ECR and 24% the criteria for partial ECR. Baseline predictors of the no-ECR group corresponded to previously identified predictors of long-term TR. Only 35 (17%) participants met the full criteria for TR at this point. Of the 97 in the no-ECR group, 18 (19%) were in an ongoing trial (p<0.001 vs ECR/partial ECR) and 21 (22%) were using the same medication over the whole follow-up year (p =.008 vs ECR /partial ECR) despite lack of significant clinical effect.

**Conclusions:**

We show that the mostly used consensus definition of TR identifies only a proportion of FEP patients without sufficient clinical and functional improvement at one year follow-up. The main reason for not meeting the criteria is a lack of two adequate antipsychotic trials at this point of time. However, only half of these were in an ongoing trial despite recommendations in clinical guidelines.

**Disclosure of Interest:**

None Declared

